# Inhibiting HMGB1-RAGE axis prevents pro-inflammatory macrophages/microglia polarization and affords neuroprotection after spinal cord injury

**DOI:** 10.1186/s12974-020-01973-4

**Published:** 2020-10-09

**Authors:** Hong Fan, Hai-Bin Tang, Zhe Chen, Hu-Qing Wang, Lei Zhang, Yu Jiang, Tao Li, Cai-Feng Yang, Xiao-Ya Wang, Xia Li, Sheng-Xi Wu, Gui-Lian Zhang

**Affiliations:** 1 Department of Neurology, The Second Affiliated Hospital of Xi’an Jiaotong University, Xi’an, 710004 Shaanxi China; 2 Institute of Neurosciences, Fourth Military Medical University, Xi’an, 710032 Shaanxi China; 3 Department of Laboratory Medicine, Xi’an Central Hospital, Xi’an Jiaotong University, 161 Xi Wu Road, Xi’an, 710003 Shaanxi China; 4 Xi’an Jiaotong University Health Science Center, Xi’an Jiaotong University, Xi’an, 710061 Shaanxi China; 5 Department of Nephrology, The Second Affiliated Hospital of Xi’an Jiaotong University, Xi’an, 710004 Shaanxi China

**Keywords:** Spinal cord injury, Macrophages/microglia, HMGB1, Polarization, RAGE

## Abstract

**Background:**

Spinal cord injury (SCI) favors a persistent pro-inflammatory macrophages/microglia-mediated response with only a transient appearance of anti-inflammatory phenotype of immune cells. However, the mechanisms controlling this special sterile inflammation after SCI are still not fully elucidated. It is known that damage-associated molecular patterns (DAMPs) released from necrotic cells after injury can trigger severe inflammation. High mobility group box 1(HMGB1), a ubiquitously expressed DNA binding protein, is an identified DAMP, and our previous study demonstrated that reactive astrocytes could undergo necroptosis and release HMGB1 after SCI in mice. The present study aimed to explore the effects and the possible mechanism of HMGB1on macrophages/microglia polarization, as well as the neuroprotective effects by HMGB1 inhibition after SCI.

**Methods:**

In this study, the expression and the concentration of HMGB1 was determined by qRT-PCR, ELISA, and immunohistochemistry. Glycyrrhizin was applied to inhibit HMGB1, while FPS-ZM1 to suppress receptor for advanced glycation end products (RAGE). The polarization of macrophages/microglia in vitro and in vivo was detected by qRT-PCR, immunostaining, and western blot. The lesion area was detected by GFAP staining, while neuronal survival was examined by Nissl staining. Luxol fast blue (LFB) staining, DAB staining, and western blot were adopted to evaluate the myelin loss. Basso-Beattie-Bresnahan (BBB) scoring and rump-height Index (RHI) assay was applied to evaluate locomotor functional recovery.

**Results:**

Our data showed that HMGB1 can be elevated and released from necroptotic astrocytes and HMGB1 could induce pro-inflammatory microglia through the RAGE-nuclear factor-kappa B (NF-κB) pathway. We further demonstrated that inhibiting HMGB1 or RAGE effectively decreased the numbers of detrimental pro-inflammatory macrophages/microglia while increased anti-inflammatory cells after SCI. Furthermore, our data showed that inhibiting HMGB1 or RAGE significantly decreased neuronal loss and demyelination, and improved functional recovery after SCI.

**Conclusions:**

The data implicated that HMGB1-RAGE axis contributed to the dominant pro-inflammatory macrophages/microglia-mediated pro-inflammatory response, and inhibiting this pathway afforded neuroprotection for SCI. Thus, therapies designed to modulate immune microenvironment based on this cascade might be a prospective treatment for SCI.

## Introduction

Spinal cord injury (SCI) initiates primary injury and a cascade of secondary injury, leading to transient or permanent functional disorder even paralysis [1]. Preservation of motor function after SCI is one of the most concerned issues during treatment and rehabilitation, which is mainly depended on the neuronal survival and myelin maintenance [2, 3]. That the relationship between neuronal or myelin loss and pro-inflammatory response after SCI has been proven by other research and our previous study [4, 5]. SCI triggers a dominant pro-inflammatory response, and resident microglia and recruited macrophages are central players in innate immune response following SCI [6, 7]. However, the pathological mechanism of the pro-inflammatory macrophages/microglia-mediated detrimental microenvironment after SCI has been not fully clarified. Thus, revealing and regulating the underlying mechanism might be a prospective strategy to promote tissue repair and functional recovery after SCI.

In trauma-induced sterile inflammation, molecules released from dead cells play pivotal roles in initialization of immune activation [8]. It was well known that apoptosis within normal physiological or pathological process is generally silent, and apoptotic cells can be swiftly removed without immune activation [9]. However, necrosis after physical trauma, infection, or toxic insult activates innate immunity [10]. Damage-associated molecular patterns (DAMPs) released from necrotic cells after injury have been proved as a key to initiate and accelerate production of pro-inflammatory mediators [11].

High mobility group box 1(HMGB1), a highly preserved nuclear DNA-binding protein, is one of the defined DAMPs [12]. As HMGB1 is expressed in all nucleated animal cells, it can be served as a powerful mediator of inflammation when passively released from necrotic cells [13]. HMGB1 has been found to participate in many immune-related diseases of the central nervous system (CNS), including traumatic brain injury, stroke, epilepsy, multiple sclerosis, and SCI [14–18]. Our previous study had demonstrated that reactive astrocytes could undergo receptor-interacting serine-threonine kinase 3 (RIP3)/mixed lineage kinase domain-like protein (MLKL)-mediated necroptosis, a programmed necrosis, and release HMGB1after SCI in mice [19]. However, it is not fully understood on how HMGB1 participated in polarization of pro-inflammatory macrophages/microglia and its related pro-inflammatory response after SCI.

The present study was designed to explore the effects and the underlying mechanism of HMGB1 on macrophages/microglia polarization, as well as its effects on neuronal and myelin loss after SCI. Both of the elevated HMGB1 concentration in serum and increased cytoplasmic HMGB1 in injured cord were found in our SCI model of rats. Our in vitro study further demonstrated that HMGB1 could polarize microglia to pro-inflammatory phenotype through receptor for advanced glycation end products (RAGE)-nuclear factor-kappa B (NF-κB) pathway. By application of glycyrrhizin, an inhibitor of HMGB1 or FPS-ZM1, an inhibitor of RAGE, we further found that inhibiting HMGB1 or RAGE decreased the numbers of pro-inflammatory macrophages/microglia while increased the numbers of anti-inflammatory cells. Furthermore, we found that inhibiting RAGE effectively enhanced neuronal survival and myelin preservation after SCI. Moreover, inhibiting both of HMGB1 and RAGE significantly improved functional recovery after SCI. Taken together, these data suggested that inhibiting HMGB1-RAGE axis prevented pro-inflammatory macrophages/microglia polarization and afforded neuroprotection after SCI in rats. It might be a prospective strategy for clinical SCI treatment based on HMGB1-RAGE axis.

## Materials and methods

### Animal grouping and treatment

A total of 150 male Sprague Dawley rats (about 250 g) were purchased from the Animal Center of Xi’an Jiaotong University. Experimental protocols of the animals in this study were approved by Animal Care and Use Committee of Xi’an Jiaotong University. SD rats were randomized into four groups: sham control group, SCI + saline group, SCI + glycyrrhizin (Sigma-Aldrich, G2137) group, and SCI + FPS-ZM1 (MedChemExpress, HY-19370) group. Rats in the sham control group received laminectomy only, while 0.5 mL of sterile saline was administrated via intraperitoneal injection (i.p.) after SCI for the SCI + saline group. According to previous researches and our pilot data [20–22], 10 mg/kg of glycyrrhizin or 1 mg/kg of FPS-ZM1 in saline was daily administered via i.p. for 14 days after SCI for the SCI + glycyrrhizin group and the SCI + FPS-ZM1 group, respectively, with the first injection immediately after SCI.

### SCI model

The SCI was established according to the modified Tetzlaff spinal cord lateral crush model as we described previously [23, 24]. Rats were anesthetized with 10% chloral hydrate (400 mg/kg), followed by laminectomy of vertebrae T8 to expose the spinal cord. The spinal cord crush model was made by the forceps (53327T, 66 Vision-Tech Co., Ltd., China) mounted on our designed mechanical device. The cord was clamped by forceps with 0.5 mm-width when fully closed, and the crush remained for 20 s. Rats in sham group underwent laminectomy only. After operation, urination was aided once a day until reestablishment of micturition reflex.

### Tissue processing

At designated time points after injury, rats were anesthetized and perfused intracardially with 200 ml of 0.01 M PBS (pH 7.4), followed by 400 ml of 4% paraformaldehyde (PFA). Two-centimeter segments of fixed tissues containing the lesion site were obtained and cryoprotected in 30% sucrose for 48 h. After embedded in OCT, both of serial transverse sections (10 μm thick) and serial sagittal sections (12 μm thick) were cut within a Leica CM1950 cryostat. The slides were collected and stored at −20 °C for staining.

### Immunohistochemistry

#### Immunoflurence

After air-dried for 2 h, slides were blocked in 0.01 M PBS containing 3% BSA and 0.1% Triton X-100 for 1 h and then incubated with primary antibodies overnight at 4 °C. The primary antibodies used in tissue staining were rabbit anti-HMGB1 (1:500, Proteintech), mouse anti-glial fibrillary acidic protein (GFAP, 1:2000, Sigma-Aldrich), rabbit anti-TLR4 (1:500, Abcam), goat anti-Iba-1 (1:500, Abcam), rabbit anti-iNOS (1:200, Abcam), and rabbit anti-Arginase-1 (1:100, Santa Cruz). Antigen retrieval was performed with citrate buffer before HMGB1-staining. After washing three times with PBS, the slides were incubated with Alexa Fluor 594-labeled or Alexa Fluor 488-labeled corresponding secondary antibodies (1:500, Jackson ImmunoResearch Laboratories) in the dark for 2 h. Nuclei were counterstained with Hoechst 33342.

#### DAB-staining

After fixation with acetone, the slides were incubated in 0.3% H_2_O_2_ solution in PBS at room temperature for 10 min to block endogenous peroxidase activity. The slides were then in turn incubated with rabbit anti-MBP (1:200, Abcam), biotinylated secondary antibody, and Avidin-Biotin Complex (ABC)-HRP conjugates. DAB substrate (0.05% DAB–0.015% H_2_O_2_ in PBS) solution was subsequently applied to reveal the color of the antibody staining. The slides were dehydrated through 4 changes of alcohol (95, 95, 100, and 100%), followed with 3 changes of xylene.

#### Immunocytoflurence

For immunocytochemistry, cells were fixed in 4% PFA for 30 min. After blocking in 3% BSA without Triton X-100 for 30 min, the cells were stained with the following primary antibodies: rabbit anti-RAGE antibody (1:200, Proteintech), rat anti-F4/80 (1:200, Bio-Rad), and rabbit anti-iNOS (1:200, Abcam). Cells were then incubated with the corresponding secondary antibodies. Sections were examined and photographed under a confocal microscope (LSM 800, Zeiss).

### Enzyme-linked immunosorbent assay (ELISA)

The concentrations of HMGB1 in serum were measured by ELISA. Rat serum was sampled, and HMGB1 levels were then determined using an ELISA kit (IBL) according to the manufacturer’s instructions. Absorbance at 450 nm was measured by multimode microplate reader (TECAN, infinite M200).

### Quantitative real-time polymerase chain reaction (qRT-PCR)

Total RNA was isolated from cultured cells or from1.5 cm length of injured cord segments (or from uninjured cord) using TRizol (Promega). After reverse transcription using a reverse transcriptase kit (TaKaRa), we performed real-time PCR for HMGB1, iNOS, IL-4, IL-10, IL-12, IL-18, CD86, CD206, TNFα, Arginase1, and Ym1 with SYBR green (TaKaRa) on Bio-Rad PCR system (Bio-Rad). The sequences of primers utilized are listed in Table 1. Gene expression levels are calculated by the 2^−ΔΔCT^ method and the data were presented normalized by GAPDH.

**Table 1:** PCR primer sequences

Gene	Forward primer(5’-3’)	Reverse primer (5’-3’)
HMGB1	GCCCATTTTGGGTCACATGG	TGCAGGGTGTGTGGACAAAA
iNOS	AGAGACGCTTCTGAGGTTCC	CTGCACCAACTCTGCTGTTC
IL-4	TCCACGGATGTAACGACAGC	TGGTGTTCCTTGTTGCCGTA
IL-10	CCTCTGGATACAGCTGCGAC	GTAGATGCCGGGTGGTTCAA
IL-12	ATCATCAAACCGGACCCACC	CAGGAGTCAGGGTACTCCCA
IL-18	ACCGCAGTAATACGGAGCAT	TCTGGGATTCGTTGGCTGTT
CD86	CGTCAAGACATGTGTAACCTGC	ACCGACTTTTTCCGGTCCTG
CD206	TCAACTCTTGGACTCACGGC	CATGATCTGCGACTCCGACA
TNFα	ACTGAACTTCGGGGTGATCG	TGGTGGTTTGCTACGACGTG
Arginase1	ACAAGACAGGGCTACTTTCAGG	ACAAGACAAGGTCAACGCCA
Ym1	AGTTTGGATCTGCCCCGTTC	TTAGGAGGGCTTCCACGAGA
GAPDH	AGTGCCAGCCTCGTCTCATA	GGTAACCAGGCGTCCGATAC

### Microglial cell culture and treatment

Microglial cells were obtained from neonatal (about 3-day-old) SD rats, as described previously [4]. Microglial cells were cultured in DMEM supplemented with 3% FBS, penicillin-streptomycin (100 U/ml–100 μg/ml) and 4 mM L-glutamine. Cells were grown on poly-L-lysine coated slides in 6-well plates. For HMGB1 and FPS-ZM1 treatment, 0.4 or 1 μg/ml of HMGB1 and 1 μM of FPS-ZM1 were added to the culture medium. At the end of treatment, total RNAs were isolated for qRT-PCR, total cellular proteins were isolated for Western-blotting, or cells were harvested for immunostaining.

### Western blotting

Whole cell lysates were prepared from 1.5-cm-long segments of injured cord containing the injury site or microglia. Protein were isolated and homogenized with ice-cold radioimmunoprecipitation assay (RIPA) buffer. Proteins were resolved on 10% sodium dodecyl sulfate-polyacrylamide gel (SDS-PAGE) gel and transferred onto a polyvinylidene difluoride (PVDF) membrane. Blots were incubated with primary antibodies for iNOS (1:500, Abcam), CD86 (1:600, Proteintech), MBP (1:1000, Milipore), RAGE (1:500, Proteintech), phospho-NF-κB p65/p-P65 (1:1000, Cell Signaling), or beta-actin (1:5000, Sigma) at 4 °C overnight. After incubation with corresponding horseradish peroxidase-conjugated secondary antibodies (1:5000, Jackson ImmunoResearch) at RT for 1 h, the bands were visualized using a Bio-Rad Image Lab system, and densitometry analysis was performed with the Image J software.

### Luxol fast blue staining

Luxol fast blue (LFB) staining was performed as described previously [4]. Serial transverse cryosections (12 μm thickness) were incubated in 0.1% Luxol fast blue (Sigma) in acidified 95% ethanol overnight at 60 °C. Differentiation and counterstaining was performed with 0.05% lithium carbonate and cresyl violet solution. LFB-stained tissue sections were examined by light microscopy and analyzed for myelin sparing.

### Nissl staining

Nissl staining was performed to assess neuronal survival. The sections were rinsed in deionized water, dipped in a warm (50 °C) solution of 1% thionine for 45 min, and differentiated with 70% alcohol for about 5 min.

### Behavioral evaluation

#### BBB scores

The Basso-Beattie-Bresnahan (BBB) scores [25] were used to evaluate functional recovery at 1 day before and 1, 3, 7, 10, 14, and 21 days after SCI. The tests were performed by two independent and blinded observers.

#### Rump-height index (RHI) assay

Rats were video-recorded from the left to the right side during walking on a runway bar (150 cm long, 8 cm wide, and 2 cm thick) before injury and at 1, 3, 7, 10, 14, and 21 dpi [26]. The RHI is defined as the height of the rump, normalized to the thickness of the beam, measured along the same vertical line. The standardized RHI (dividing post-injury value by pre-injury value) was introduced to minimize the variations of pre-surgery RHI of each animal as we used previously [27].

### Image analysis

The sections of GFAP staining, Nissl staining, and LFB staining were captured and examined using a fluorescence microscope (BX51, Olympus). The lesion area delineated by GFAP immunohistochemistry was calculated by Photoshop CS3 and converted to the physical size based on microscope calibration. The other immunostained sections were photographed under a confocal microscope (LSM 800, Zeiss).

For cell counting, all of the positive cells in the defined area in randomly chosen slide were counted. Then, calibration of the profile numbers was adopted to minimize the unbiased data as recommended [28, 29].

### Statistics

Quantification was performed by researchers who were blinded to the experiment design. Data are expressed as mean ± standard error of the mean (SEM). The Student’s t-test was used for comparisons between two groups. For multiple comparisons, the data were analyzed by one-way ANOVA followed by Tukey post-hoc tests. Assessment of BBB scales and RHI was analyzed by two-way RM ANOVA. P <0.05 was considered statistically significant.

## Results

### Increased expression and release of HMGB1 after SCI

**Fig. 1:**
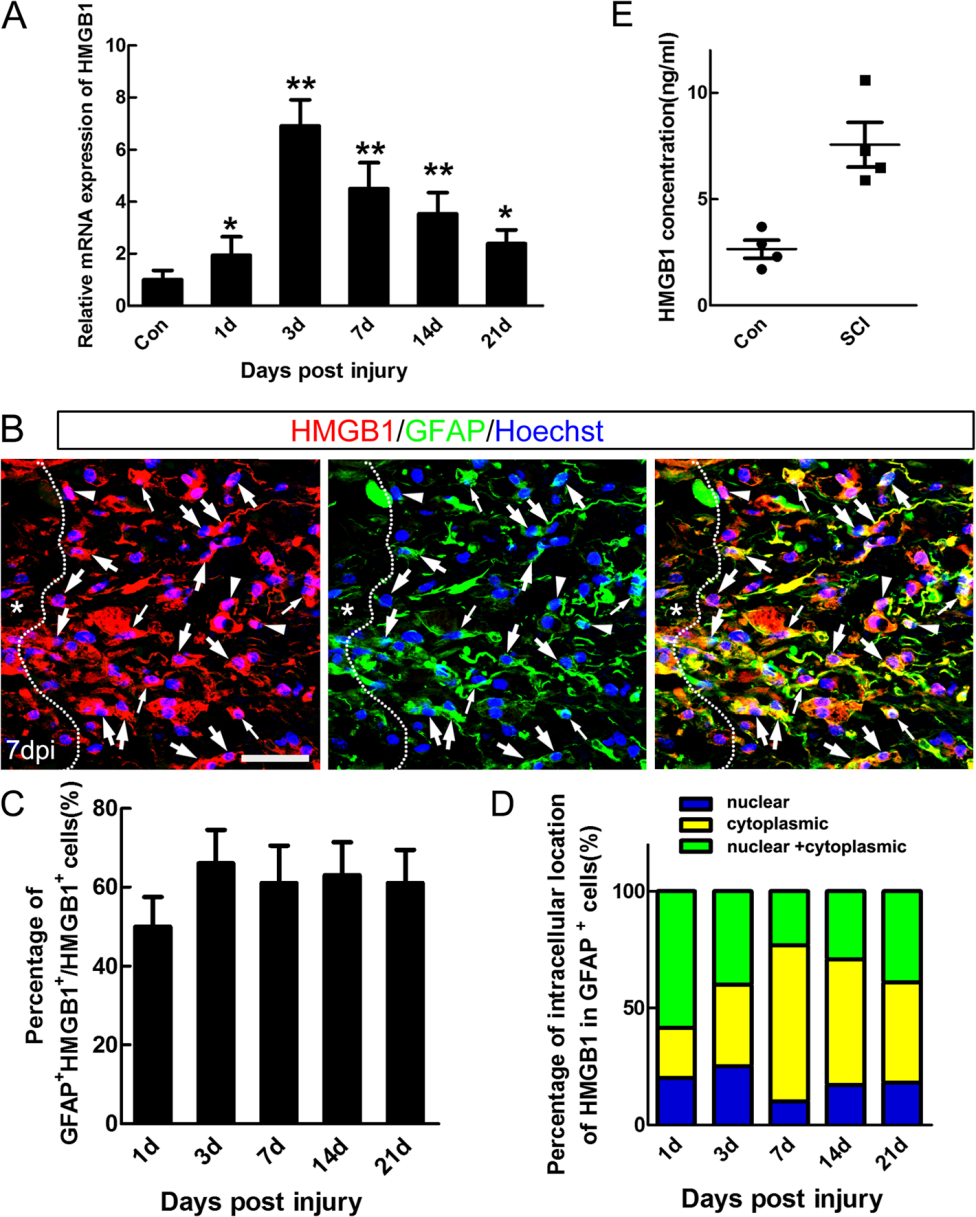
HMGB1 Expression after SCI. **a** Temporal pattern of HMGB1 mRNA in spinal cord of sham-operated and SCI rats. Note that the mRNA level of HMGB1 was significantly increased from 3 to 21 dpi. Results were expressed as mean ± SEM of 3 rats, *P < 0.05, **P < 0.01. **b** Immunohistochemistry of HMGB1. Note that all the cells around the epicenter were labeled with HMGB1-positive signal, with different intracellular localization (nuclear indicated by arrowheads, cytoplasmic indicated by thick arrows, and both cytoplasmic and nuclear indicated by thin arrows). Asterisks indicate the epicenter. Scale bar = 80 μm. **c**–**d** Quantification of intracellular localization of HMGB1. About 60% of the HMGB1-positive cells were GFAP-positive. Note that percentage of cytoplasmic HMGB1 in GFAP-positive cells reached peak (67.0 ± 7.56%) at 7 dpi, and lowest percentage (10.2 ± 1.81%) of nuclear HMGB1 at 7 dpi. **e** Elevated HMGB1 serum levels in 4 rats 3 days after SCI compared with sham-operated controls, *P < 0.05

We first examined the expression changes of HMGB1 after SCI, one potential mediator from the necrotic core of injured cord, and the data of qPCR showed that the mRNA level of HMGB1 increased significantly from 1 to 21 dpi, with the highest level at 3 dpi (*P < 0.05, **P < 0.01, Fig. 1a). Immunohistochemistry detected cytoplasmic, nuclear, and both cytoplasmic and nuclear HMGB1-immunoreactivity around lesion center (Fig. 1b), and quantification showed that approximately 60% of the HMGB1-positive cells were GFAP-positive (Fig. 1c), which was consistent with our previous result in mice of SCI [19]. Further data showed that percentage of cytoplasmic HMGB1 in GFAP-positive cells gradually increased and reached peak (67.0 ± 7.56%) at 7 dpi, percentage of both cytoplasmic and nuclear HMGB1-immunoreactivity gradually decreased from 1 dpi, and lowest percentage (10.2 ± 1.81%) of nuclear HMGB1 was found at 7 dpi (Fig. 1d). As it was reported that HMGB1 functions as a DAMP to induce a pro-inflammatory response when it is passively released from the necrotic cells [30], we tested the serum HMGB1 levels by ELISA after SCI. The result showed that the concentration of HMGB1 was significantly increased in serum at 3 days after SCI, when compared to sham-operated controls (Fig. 1e). These data suggested that the released HMGB1 might be a candidate DAMP to contribute to the persistent pro-inflammatory response after SCI.

### HMGB1 induced pro-inflammatory polarization of microglial cells

**Fig. 2:**
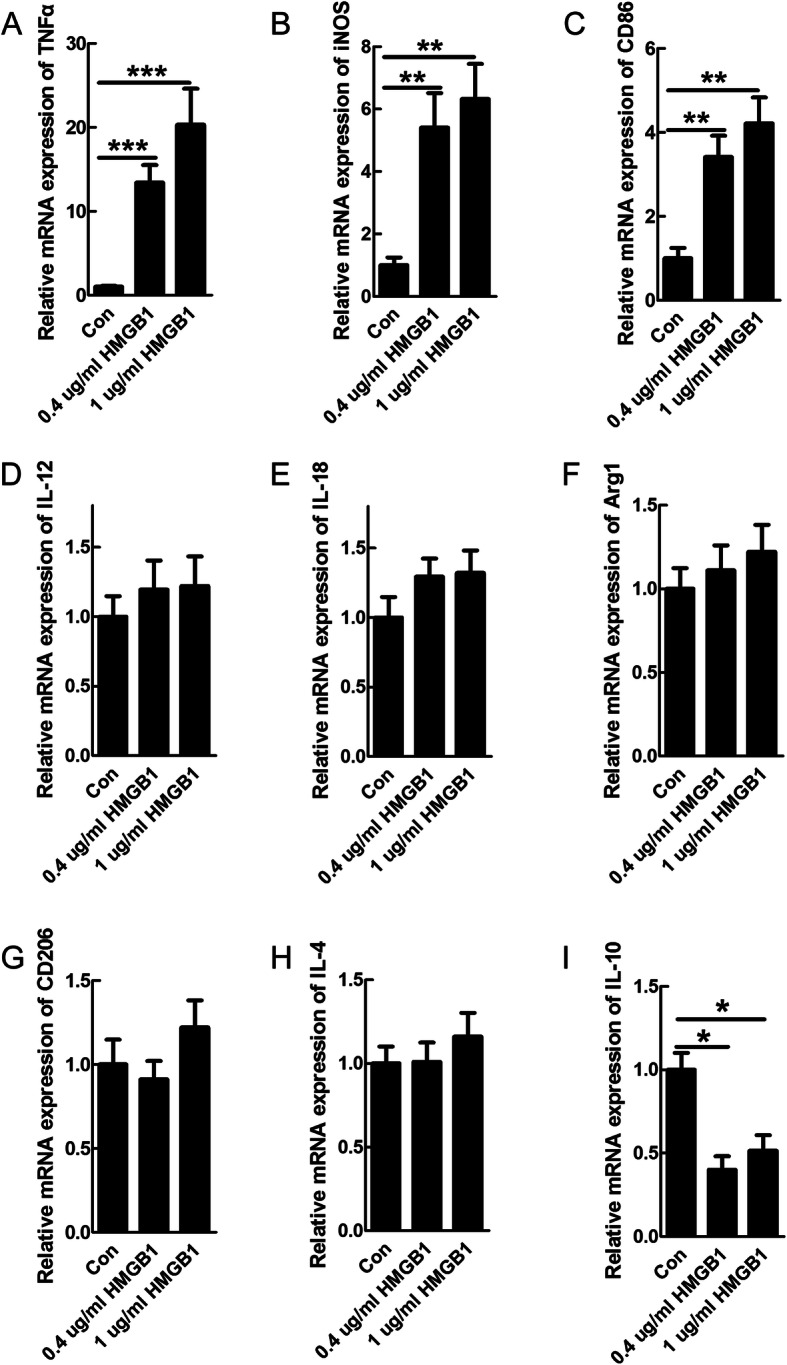
HMGB1 increased the expression of pro-inflammatory markers in the microglia. The mRNA expression of pro-inflammatory markers of TNFα (**a**), iNOS (**b**), CD86 (**c**), and IL-12 (**d**) was significantly increased in microglia 24 h after recombinant HMGB1 treatment, except for no change of IL-18 (**e**). No significant changes in anti-inflammatory markers of Arg1, CD206, and IL-4 after HMGB1 treatment (**f**–**h)**, except for a decrease of IL-10 (**i**). Results are presented as mean ± SEM. *N* = 3, *P < 0.05, **P < 0.01, ***P < 0.001

Although HMGB1 was reported to induce pro-inflammatory response after SCI [31], there still lacks direct evidence of HMGB1 participating in polarization of pro-inflammatory microglia. To determine the effect of HMGB1 on polarization of microglia, we examined the mRNA levels of pro-inflammatory or anti-inflammatory phenotypic markers in primary-cultured microglia upon HMGB1 treatment. The data showed that mRNA levels of TNFα, iNOS, and CD86 were significantly increased in microglia treated with 0.4 or 1 μg/ml HMGB1, compared to cells under normal condition (**P < 0.01, ***P < 0.001, Fig. 2a–c), while levels of IL-12 and IL-18, another two pro-inflammatory markers, showed no significant changes upon HMGB1 treatment (Fig. 2d–e). Nevertheless, no significant changes in anti-inflammatory markers Arg1, CD206, and IL-4 were observed after HMGB1 treatment (Fig. 2f–h), except for a decrease of IL-10 (*P < 0.05, Fig. 2i). The above data provided direct evidence for HMGB1 in polarizing microglia to pro-inflammatory state.

### RAGE mediated the HMGB1-induced pro-inflammatory polarization of microglia

**Fig. 3:**
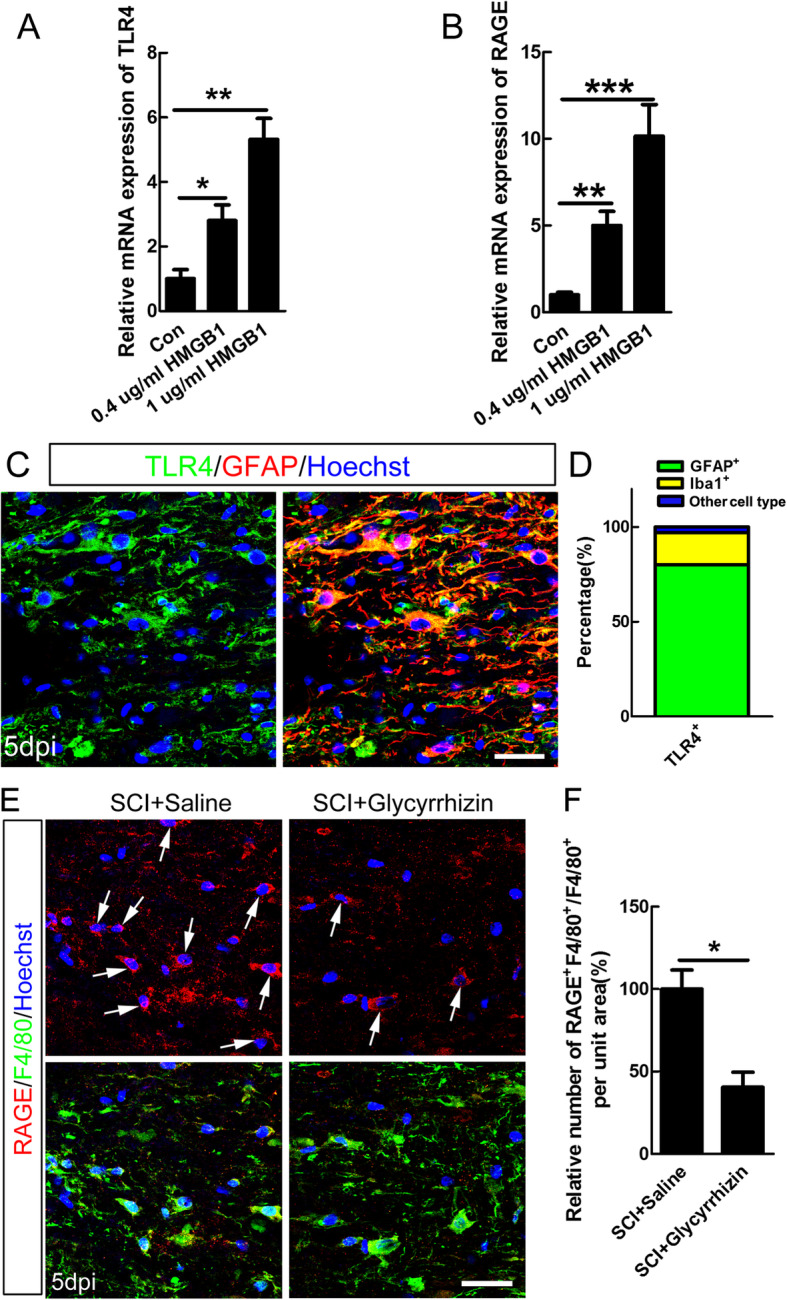
HMGB1 activated TLR4 and RAGE in vitro, and SCI induced the expression of RAGE in macropgahes/microglia. The mRNA expression of TLR4 (**a**) and RAGE (**b**) was significantly increased in microglia at 24 h after 0.4 or 1 μg/ml HMGB1 treatment. (**c**–**d**) Double-staining of GFAP/TLR4 and quantification of TLR4-positive cell types at 5 dpi. Note that about 80% TLR4-positive cells were GFAP-positive, while 17% were Iba-1 positive. Scale bar = 50 μm. **e** Representative images of double-staining of RAGE and F4/80 in injured cord of saline- or glycyrrhizin-treated rats. Scale bar = 50 μm. **f** Quantification of the relative percentage of RAGE-positive cells in F4/80-positive cells. *N* = 4, *P < 0.05

Considering that HMGB1 could function as a DAMP through receptors TLR4 or RAGE during distinct conditions [32], we then investigated the specific receptor involved in pro-inflammatory polarization of macrophages/microglia upon HMGB1 treatment. The data of qPCR showed that HMGB1 increased both mRNA levels of TLR4 and RAGE in microglia (Fig. 3a–b). Although that immunostaining showed that TLR4 was mainly expressed by GFAP-positive cells 5 dpi, with only 17% of TLR4-positive were Iba-1-positive (Fig. 3c–d, Additional file 1: Figure S1), the possibility that activation of TLR4 on astrocytes might influence macrophage phenotype through a paracrine method could not be excluded. Further, immunohistochemistry showed that the positive staining of RAGE was mainly co-localized with F4/80-positive macrophages/microglia after SCI. Furthermore, we found that glycyrrhizin, an inhibitor of HMGB1 could decrease the percentage of RAGE-positive cells in F4/80-positive cells after SCI (Fig. 3e–f). The above results suggested that RAGE was a possible receptor for pro-inflammatory polarization of microglia upon HMGB1 treatment.

**Fig. 4:**
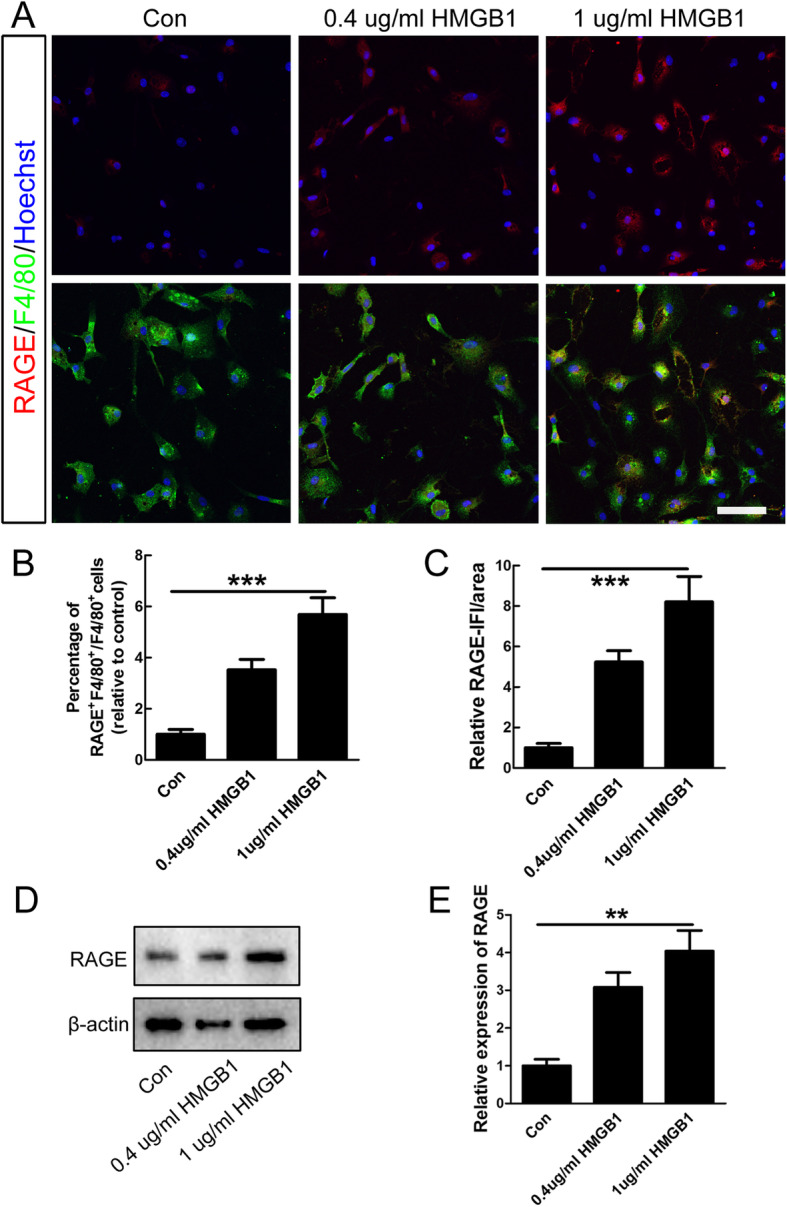
HMGB1 induced RAGE expression in microglia. **a** Representative images of double-staining of RAGE and F4/80 in microglia under normal condition or HMGB1 treatment. Scale bar = 50 μm. **b**–**c** Quantification of the numbers of RAGE-positive cells and the IFI/area of RAGE. Note that both the percentage of RAGE-positive cells and IFI of RAGE were significantly increased after HMGB1 treatment. *N* = 3, ***P < 0.001. IFI, immunofluorescence intensity. **d**–**e** Quantification of the protein expression levels of RAGE in control and HMGB1-treated group. β-actin was used as a loading control. Data were expressed as mean ± SEM. *N* = 3, **P < 0.01

Our immunocytochemistry data revealed that the numbers of RAGE-positive cells and its immunofluorescence intensity (IFI) were significantly increased in microglia upon HMGB1 treatment in vitro (***P < 0.001, Fig. 4a–c). The data of western blot also showed that HMGB1 induced expression of RAGE in microglia (**P < 0.01, Fig. 4d–e). As 1 μg/ml of HMGB1 exerted a stronger inducible effect on expression of pro-inflammatory markers and RAGE, this concentration of HMGB1 was adopted for further experiments.

**Fig. 5:**
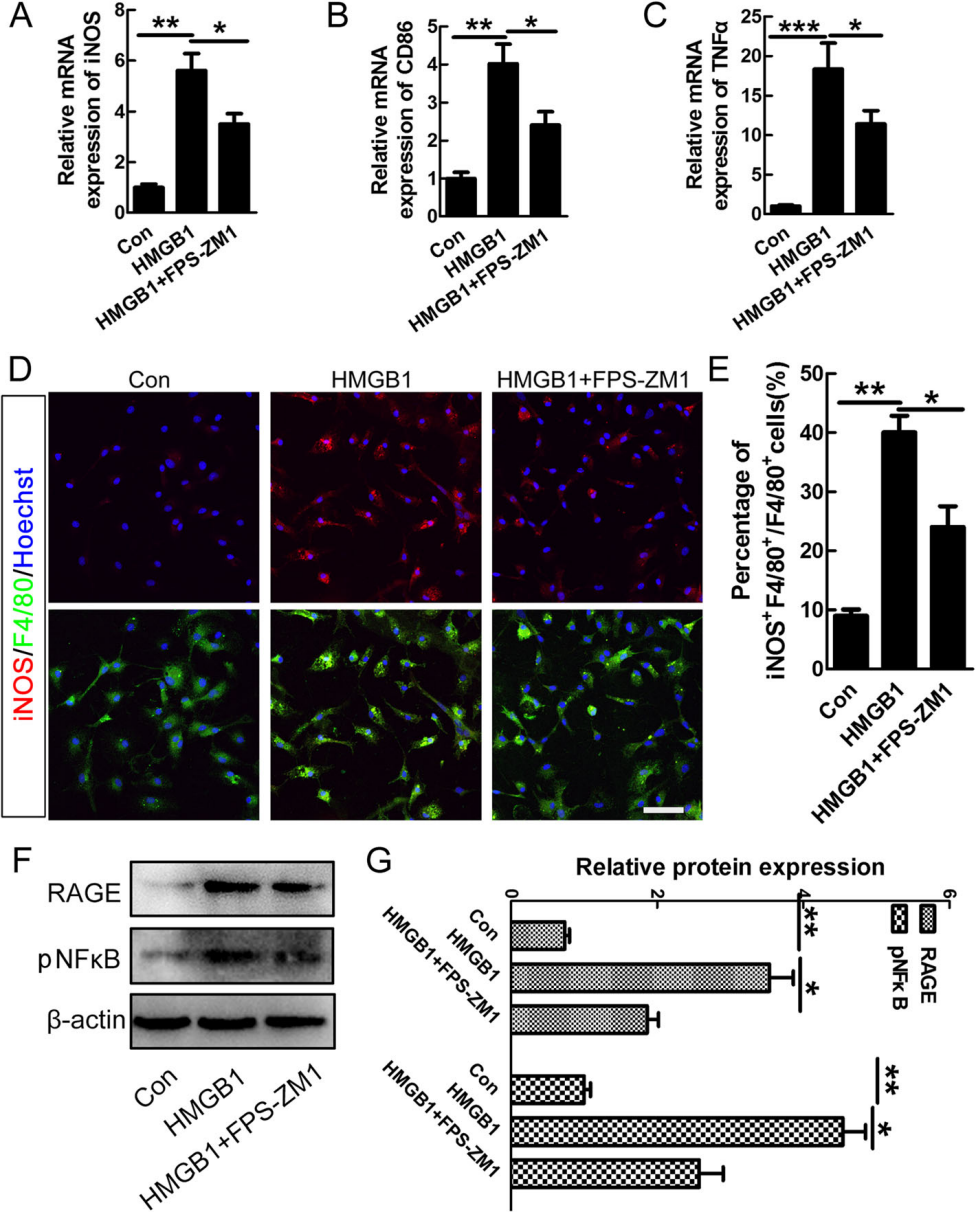
Inhibiting RAGE-suppressed HMGB1- or SCI-induced pro-inflammatory polarization of macrophages/microglia. FPS-ZM1 (a RAGE-specific blocker) significantly reversed the increased mRNA of iNOS (**a**), CD86 (**b**), and TNFα (**c**) induced by HMGB1. *N* = 3, *P < 0.05, **P < 0.01, ***P < 0.001. **d** Representative images of double-staining of iNOS and F4/80 in microglia under normal condition, HMGB1 or HMGB1plus FPS-ZM1 treatment. Scale bar = 30 μm. **e** Quantification of the percentage of iNOS-positive cells in F4/80-positive cells. Note that FPS-ZM1 significantly inhibited the increased number of iNOS-positive cells induced by HMGB1. *N* = 3, *P < 0.05, **P < 0.01. **f**–**g** Quantification of the expression levels of RAGE and p-NF-κB in microglia under different conditions. β-actin was used as a loading control. Data were expressed as mean ± SEM. *N* = 3, *P < 0.05, **P < 0.01

We further applied FPS-ZM1, a RAGE-specific blocker to examine whether RAGE was involved in HMGB1-induced pro-inflammatory polarization of the microglia. Our data showed that FPS-ZM1 significantly reversed the increased mRNA of iNOS, CD86, and TNFα induced by HMGB1 (*P < 0.05, **P < 0.01, ***P < 0.001, Fig. 5a–c). The result of immunostaining also showed that FPS-ZM1 significantly decreased the numbers of iNOS-positive cells induced by HMGB1 (*P < 0.05, **P < 0.01, Fig. 5d–e). The data of western blot further showed that FPS-ZM1 could reduce the expression of RAGE and its downstream molecule p-NF-κB induced by HMGB1 (*P < 0.05, **P < 0.01, Fig. 5f–g). The above data indicated that HMGB1-induced pro-inflammatory polarization of microglia was at least partially through RAGE-NF-κB pathway.

### Inhibiting HMGB1-RAGE axis influenced the polarization of macrophages/microglia after SCI

**Fig. 6:**
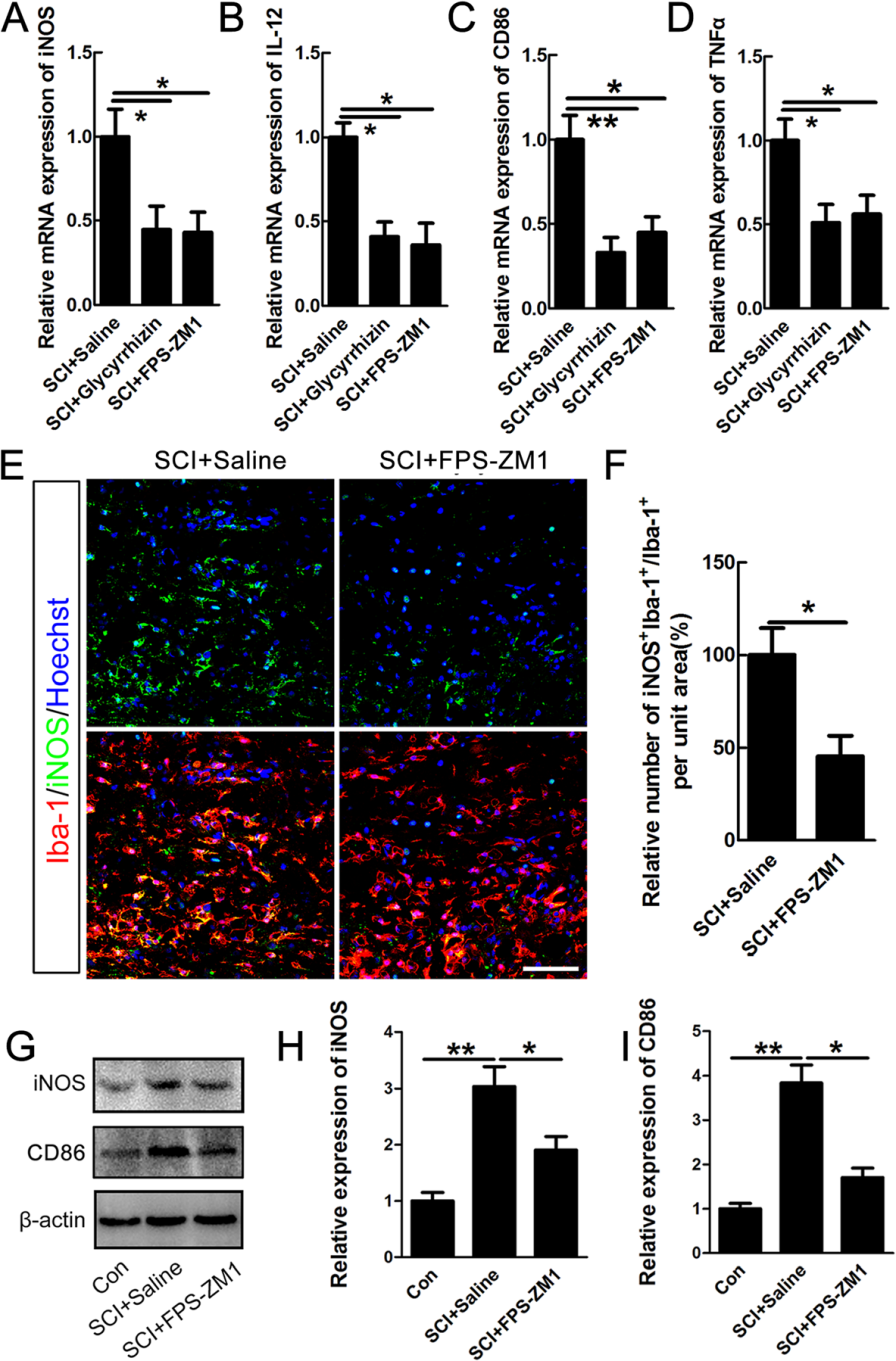
Inhibiting HMGB1or RAGE reduced the numbers of pro-inflammatory macrophages/microglia after SCI. Quantification of pro-inflammatory mRNA of iNOS (**a**), IL-12 (**b**), CD86 (**c**), and TNFα (**d**) in saline-, glycyrrhizin-, or FPS-ZM1-treated rats at 14 dpi. Results are presented as mean ± SEM. *N* = 3/group, *P < 0.05. **e** Representative images of double-staining of iNOS and Iba-1 in saline- or FPS-ZM1-treated rats at 14 dpi. Scale bar = 80 μm. **f** Quantification of iNOS-positive microglia/macrophages in the bilateral areas 200 μm rostral and caudal to the lesion site. Notice the decrease of iNOS-positive microglia/macrophages in FPS-ZM1-treated rats. Results are mean ± SEM. *N* = 5/group, *P < 0.05. **g**–**i** Quantification of the expression levels of iNOS and CD86 in sham group, saline-, or FPS-ZM1-treated rats at 14 days after SCI. *N* = 5/goup, *P < 0.05, **P < 0.01

Based on the above in vitro data, we applied glycyrrhizin and FPS-ZM1 to determine the effects of HMGB1-RAGE inhibition on polarization of macrophage/microglia after SCI. The data showed that both glycyrrhizin and FPS-ZM1 significantly decreased the mRNA levels of pro-inflammatory markers of iNOS, IL-12, CD86, and TNFα (*P < 0.05, Fig. 6a–d) at 14 days after injury. Further, immunostaining showed that the number of iNOS-expressing macrophages/microglia in bilateral areas 200 μm rostral and dorsal to lesion center was significantly decreased in FPS-ZM1-treated rats (*P<0.05, Fig. 6e-f), while no significant differences in total number of macrophage/microglia between saline and FPS-ZM1 group (data not shown). Furthermore, the data of western blot showed that FPS-ZM1 inhibited the injury-induced pro-inflammatory polarization, shown by lowered levels of iNOS and CD86 (*P<0.05, **P<0.01, Fig. 6g-i).

Meanwhile, we tested the effect of HMGB1-RAGE inhibition on anti-inflammatory polarization. The data showed that both glycyrrhizin and FPS-ZM1 significantly increased the mRNA levels of Arginase1, Ym1, IL-4, and IL-10 (Additional file 2: Figure S2A–D). The immunohistochemical data further revealed that the Arginase1-positive macrophages/microglia in the epicenter was significantly increased in the FPS-ZM1 group (Additional file 2: Figure S2 E–F). In the sham-operated rats, there were none of the iNOS- or Arginase1-positive cells (data not shown). The above results demonstrated that HMGB1 or RAGE inhibition could suppress macrophages/microglia polarized to pro-inflammatory phenotype, while promote anti-inflammatory polarization after SCI.

### Inhibiting RAGE decreased lesion size and neuronal death after SCI

**Fig. 7:**
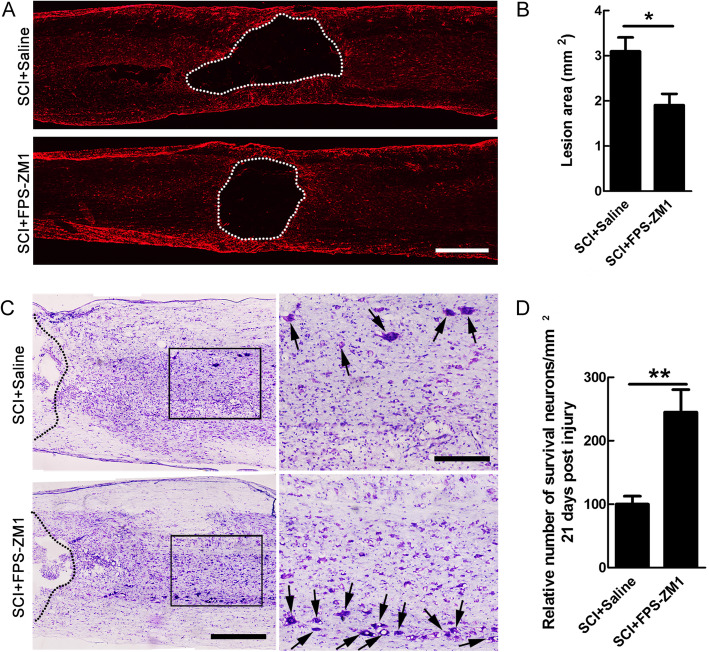
The effects of FPS-ZM1 treatment on lesion area and neuronal survival after SCI. **a** Dashed lines indicate the lesion border defined by GFAP immunoreactivity in saline- or FPS-ZM1-treated rats at 21 dpi. Scale bar = 500 μm. **b** Quantification of lesion area. *N* = 6/group, *P < 0.05. **c** A representative Nissl-stained section of saline- or FPS-ZM1-treated rats at 21 dpi. Broken lines mark the lesion borders. Arrows indicate Nissl-stained neurons. Scale bar = 600 μm in the left lower-magnification pictures and 100 μm in the right higher-magnification pictures from the boxes of left. **d** Quantification of the numbers of Nissl-stained neurons at 21 dpi. Note that the numbers of Nissl-stained neurons was significantly increased by FPS-ZM1 treatment in rostral and caudal areas of 600 μm adjacent to lesion. *N* = 6/group, **P < 0.01

The special pro-inflammatory macrophages/microglia-mediated detrimental response after SCI was closely related to the degree of secondary injury [33]. Considering that inhibiting HMGB1could ameliorate the proimmune environment [34], we subsequently assessed the effects of HMGB1-RAGE inhibition on lesion cavity and neuronal survival. Immunohistochemistry for GFAP was performed to evaluate the lesion size, and the result showed that FPS-ZM1 significantly reduced the lesion area at 21 dpi (*P < 0.05, Fig. 7a–b). Furthermore, the data of Nissl staining showed that the number of Nissl-stained neurons in the area of 600 μm away from each side of the lesion was increased by 1.8 times in FPS-ZM1-treated rats at 21 dpi (**P < 0.01, Fig. 7c–d).

### Inhibiting RAGE reduced myelin loss after SCI

**Fig. 8:**
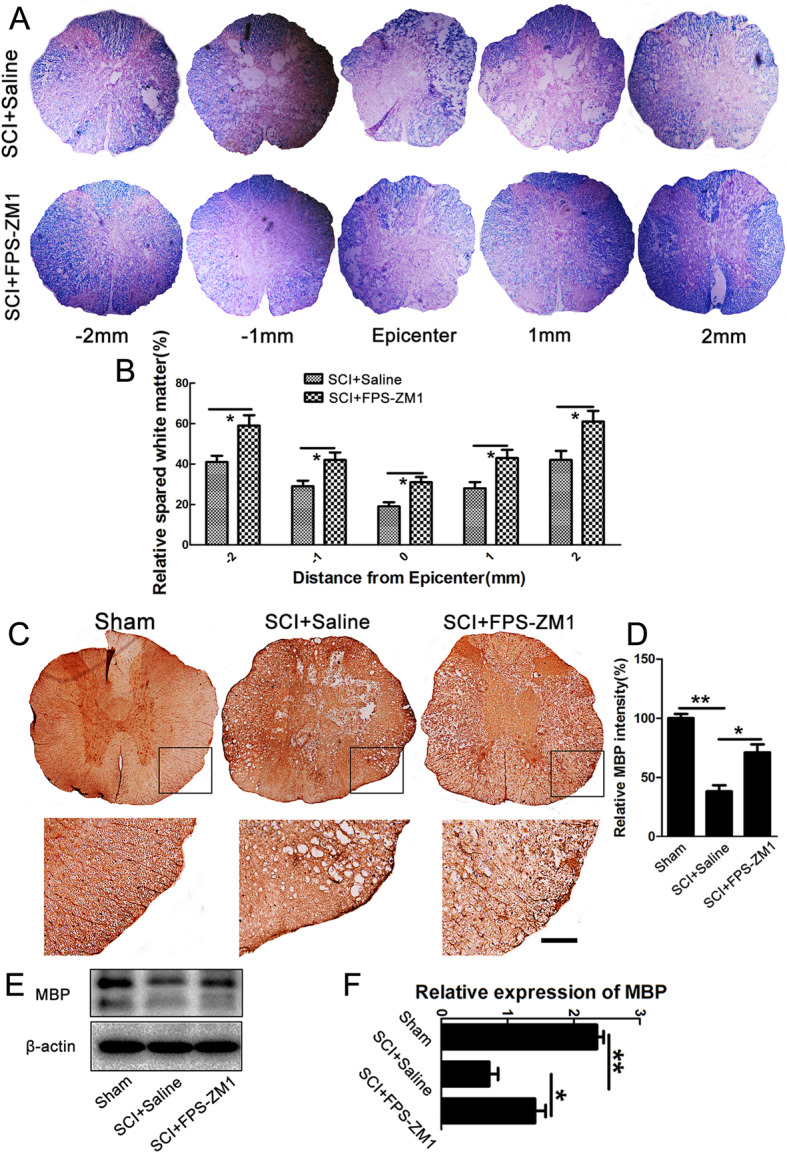
Inhibiting RAGE by FPS-ZM1 reduced myelin loss after SCI. **a** Spinal cords at 21 days after injury were processed for Luxol fast blue in saline- or FPS-ZM1-treated rats. Transverse cryosections were selected 1000 μm, 2000 μm rostral and caudal to the lesion site, and the epicenter. **b** Quantification of spared myelin at 1000 μm, 2000 μm rostral and caudal to the lesion site, as well as at the epicenter. Data were expressed as mean ± SEM of 6 rats, *P < 0.05. **c** DAB staining of MBP in transverse sections at 21 dpi. Scale bar = 100 μm. **d** Quantification of MBP intensity. Note that FPS-ZM1 attenuated the reduction of MBP intensity in the white matter after injury. Data were expressed as mean ± SEM of 6 rats, *P < 0.05, **P < 0.01. **e**–**f** Quantification of the protein expression level of MBP in sham group, saline-, or FPS-ZM1-treated rats at 21 days after SCI. *N* = 6/group, *P < 0.05, **P < 0.01

Besides neuronal survival, we also observed myelin preservation, which was another pivotal factor for locomotion recovery after SCI. LFB staining, immunostaining, and western blot were performed to assess the myelin loss. Data of LFB staining showed that FPS-ZM1 significantly decreased myelin loss at 2000 μm rostral and caudal to the injury site, as well as at the epicenter (*P < 0.05, Fig. 8a–b). Moreover, the intensity of MBP-positive myelin was significantly increased in FPS-ZM1-treated rats (**P < 0.01, *P < 0.05, Fig. 8c–d). The data of western blot further confirmed that the spared myelin was significantly increased after FPS-ZM1 treatment (**P < 0.01, *P < 0.05, Fig. 8e–f). In addition, our data from 5-bromo-2’-deoxyuridine (BrdU) incorporation assay showed that FPS-ZM1 had no significant effect on oligodendrocyte regeneration after SCI (data not shown). The above data indicated that inhibiting RAGE markedly reduced the extent of myelin loss after SCI.

### Inhibiting HMGB1-RAGE axis improved locomotor recovery after SCI

**Fig. 9:**
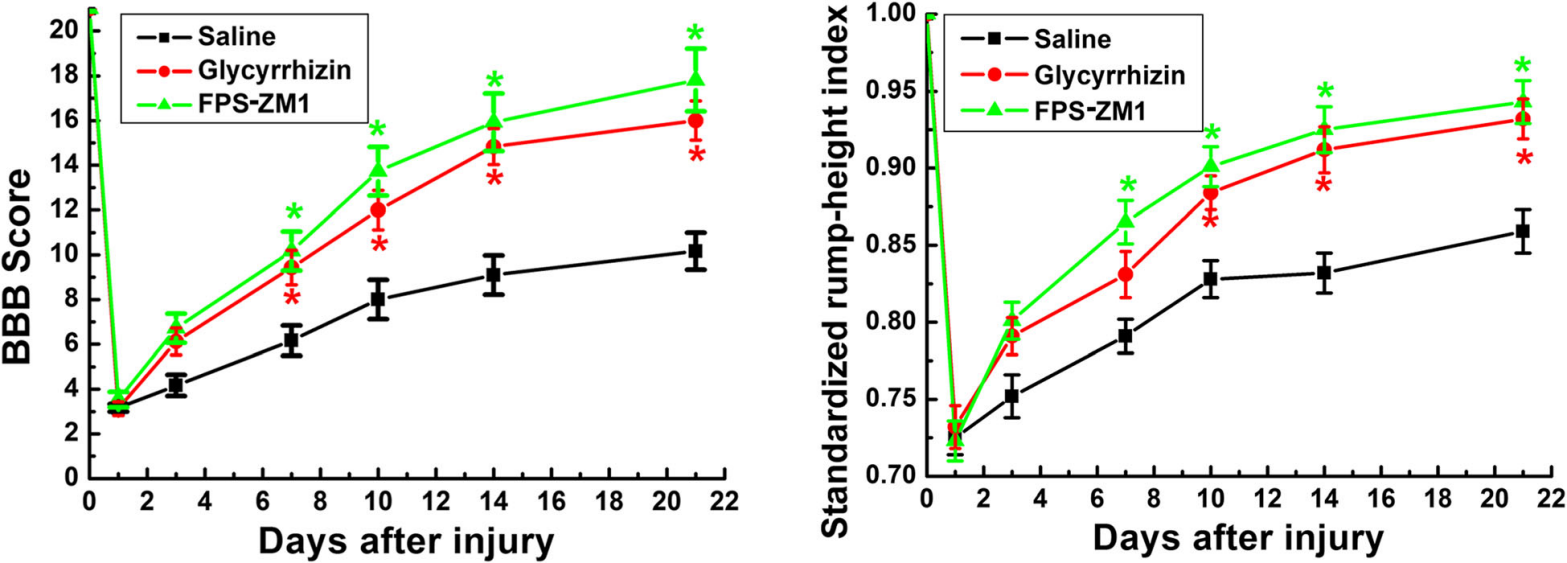
Locomotion recovery improvement by glycyrrhizin or FPS-ZM1 treatment. **a** BBB scores at 1, 3, 7, 10, 14, and 21 days after SCI. The average BBB scores in glycyrrhizin- or FPS-ZM1-treated group were significantly higher than those in saline group at 7, 10, 14, and 21 days after injury. **b** SRHI values at 1, 3, 7, 10, 14, and 21 days after SCI. *N* = 6/group, *P < 0.05

To test whether inhibiting HMGB1 or RAGE is beneficial to the locomotor recovery after SCI, BBB scoring and RHI assay were performed before injury and at 1, 3, 7, 10, 14, and 21 days after SCI. Compared with saline control, the glycyrrhizin or FPS-ZM1-treated rats showed significant higher BBB scores from 7 dpi (*P < 0.05, Fig. 9a). Similarly, the glycyrrhizin or FPS-ZM1-treated rats showed higher lift of the hind limbs from 7 dpi as evaluated by the RHI assay (*P < 0.05, Fig. 9b).

## Discussion

Better and deeper understanding of the pathological mechanism after SCI is crucial for developing new treatment. The pro-inflammatory macrophages/microglia-mediated pro-inflammatory response dominates the innate immune responses after SCI [6], but the mechanisms underlying this special process have been not fully elucidated. In the present study, we found that inhibiting the HMGB1-RAGE axis effectively prevented pro-inflammatory polarization of macrophages/microglia, decreased neuronal and myelin loss, and improved functional recovery after SCI.

Although it was found that HMGB1-RAGE does not aggravate neuronal inflammation [35], others demonstrated that inhibiting HMGB1 or RAGE could suppress the inflammation after injury [31, 34, 36–38]. The different results may be due to the different responses of neural cells to HMGB1 treatment and the different RAGE inhibitors used in different researches. However, the specific mechanism of HMGB1 or RAGE inhibition targeted on macrophages/microglia polarization had never been stated.

The data from genome-wide transcriptomics and proteomics indicated that the polarization of microglia and macrophage was not exactly the same [39]; we thus employed the terminology as pro-inflammatory (M1-like) or anti-inflammatory (M2-like) phenotype as recommended [40]. Previous studies indicated the pro-inflammatory polarization of macrophages upon HMGB1 challenge [31], while HMGB1 was also reported to enhance the protumoral activities of anti-inflammatory macrophages [41]. However, the HMGB1-RAGE axis in polarization of macrophages/microglia after SCI has never been clearly defined. In this study, we first demonstrated that HMGB1 polarized pro-inflammatory microglia through RAGE-NF-κB pathway. Besides pro-inflammatory phenotype, we observed the effects of inhibiting HMGB1-RAGE on anti-inflammatory polarization of macrophages/microglia as shown in Additional file 2. It will be an independent project to investigate its mechanism, because none of the molecular determinants of pro-anti-inflammatory polarization such as the PPAR, KLF, IRF, STAT, NF-κB, and HIF families and miRNAs [42] could be excluded before exploration.

Besides the effects of HMGB1-RAGE inhibition on polarization of macrophages/microglia, we also observed its neuroprotective effects with focus on neuronal survival and myelin loss. Although it was reported that anti-RAGE antibody do damage to neuronal survival [43], we demonstrated that RAGE blockade could decrease neuronal loss by its inhibitor, FPS-ZM1. We proposed that the different effects might be attributed to the different inhibitors of RAGE. In addition, blocking RAGE was found to promote oligodendrocyte autophagy in spinal cord injury [44], which was similar with the results of the reduced myelin loss in our study. In this study, the direct function of RAGE inhibition on differentiation of oligodendrocyte precursor was precluded; we thus proposed that the reduced myelin loss by FPS-ZM1 in this study was probably due to the improved immune microenvironment of SCI. Remarkable functional recovery on BBB scale were observed from 8 to 9 to 18 in glycyrrhizin- or FPS-ZM1-treated rats, and we speculated that it is probably due to the increased numbers of neurons and spared myelin, with other mechanisms cannot be excluded.

HMGB1 has been demonstrated to be localized in the nucleus of astrocytes, microglia, neurons, and oligodendrocytes in naive spinal cord [45, 46], and its cellular localization was almost unchanged except for the cytoplasmic localization in Mac-1-positive macrophages in mice after SCI [31]. In this study, we observed that cytoplasmic HMGB1 was mainly localized in GFAP-positive astrocytes after SCI in rats, which was consistent with our previous result in SCI of mice [19]. In addition, cytoplasmic HMGB1 has been reported to be expressed by neurons after traumatic brain injury [47]. We speculated that the differences might be attributed to the species diversity and the different injury model. It has been definitively proved that HMGB1 functions as a DAMP to alert the innate immune system only when it is passively released by necrotic cells but not from cells undergoing apoptosis [13, 48]. As our previous study demonstrated that necroptotic astrocytes could release HMGB1 [19, 49], we speculated that the elevated serum HMGB1 was mostly originated from the HMGB1-positive astrocytes, which might undergo necroptosis. We will test and verify this hypothesis by application of RIP3 and MLKL knock-out mice in future. In addition, it is important to prove antibody specificity first for immunohistochemistry as the guidelines showed [50]. Besides the validation by the manufacturer or the previous study [51], the specificity of antibodies of HMGB1, RAGE, and TLR4 was verified by western blot as shown in Additional file 3, and the full gels of other antibodies are shown in Additional file 4.

In recent years, several small molecules, derived either from natural sources or chemical synthesis explored as inhibitors of HMGB1, showed significant therapeutic importance [52]. Glycyrrhizin was one of the most widely used natural inhibitors, and studies have demonstrated that glycyrrhizin protected the liver, brain, and cardiovascular injury in experimental animals [53–55]. It was also reported that glycyrrhizin attenuated the transient spinal cord ischemic injury in rats via reducing inflammatory cytokines and inhibiting the release of HMGB1 [36]. In this study, the inhibition of release of HMGB1 was not found by glycyrrhizin treatment after SCI (data not shown), and we proposed that glycyrrhizin could inhibit the receptor activation of HMGB1 as shown by the decreased percentage of RAGE-positive cells in F4/80-positive cells after SCI. Besides the small molecules, anti-HMGB1 monoclonal antibody (mAb) had been demonstrated to neutralize the released HMGB1, prevent the inflammation cascade, and afford a therapeutic effect in ischemic brain injury, traumatic brain injury, Parkinson’s disease, and SCI [14, 56–59]. One research showed that acute intraperitoneal use of HMGB1 neutralizing antibody failed to provide protection after SCI [31], which suggested that small molecule inhibitors of HMGB1 such as glycyrrhizin may be preferable to blocking antibodies since these molecules can bypass the blood-spinal cord barrier more effectively. On the other hand, beneficial effects of anti-HMGB1 mAb for SCI depended on a proper time window for drug administration as demonstrated previously [59]. In addition, it was reported that prior treatment with anti-HMGB-1 antibody enhanced human neural stem cell transplantation-mediated functional recovery after SCI, and this effect was mediated by increased synaptic connectivity between increased preserved host neurons and transplant-derived neurons [60], which was similar as increased neuronal survival by FPS-ZM1 in this study.

Although at least 14 different receptor systems have been reported to be engaged by extracellular HMGB1, only TLR4 and RAGE have so far been extensively studied in carefully defined molecular systems and confirmed in a substantial number of studies to act as specific HMGB1 receptors [32]. We thus examined the changes of these two receptors, and our data of qPCR and immunostaining suggested that TLR4 was unlikely to be involved in HMGB1-induced pro-inflammatory polarization of microglia. By application of FPS-ZM1, we demonstrated that RAGE contributed to the pro-inflammatory polarization of microglia upon HMGB1 treatment. Nevertheless, RAGE was reported to be expressed by neurons, glia, and endothelial cells [61–64]. We therefore cannot exclude the possibility that the activation of RAGE in neurons or endothelial cells attributed to the neuroprotective effect of HMGB1 inhibition.

## Conclusions

This study demonstrated that inhibiting HMGB1-RAGE axis prevented pro-inflammatory macrophages/microglia polarization and afforded neuroprotection after SCI in rats. Thus, therapies targeting HMGB1-RAGE axis might be a prospective clinical treatment for SCI.

## Supplementary information

**Additional file 1: Figure S1.** Double-staining of Iba-1/TLR4 at 5dpi. Note that about 17% TLR4-positive cells were Iba-1 positive. Scale bar = 50 μm.

**Additional file 2: Figure S2.** Inhibiting HMGB1 or RAGE increased the numbers of anti-inflammatory macrophages/microglia after SCI. Quantification of anti-inflammatory-associated mRNA transcripts of Arginase1 **(A)**, Ym1 **(B)**, IL-4 **(C)**, IL-10 **(D)** in saline-, FPS-ZM1- or Glycyrrhizin-treated rats at 14 dpi. Results are presented as mean ± SEM of n=3, *P<0.05. **(E)** Double-staining of Arginase1 and Iba-1 in saline- or FPS-ZM1-treated rats at 14 dpi. Scale bar = 80 μm. **(F)** Quantification of the numbers of Arginase1-positive microglia/macrophages in lesion epicenter. Results are presented as mean ± SEM. N = 5/group, *P<0.05.

**Additional file 3: Figure S3.** Western blot of HMGB1, RAGE, and TLR4. The specificity of antibodies of HMGB1, RAGE and TLR4 was verified by western blot. Note that the antibodies (HMGB1, RAGE, and TLR4) recognize only one antigen respectively, and each band is of the appropriate molecular weight.

**Additional file 4: Figure S4.** Full gels of RAGE, pNF-κB, iNOS, CD86 and β-actin.

## Data Availability

The datasets generated and/or analyzed in the current study are available from the corresponding author on reasonable request.

## Abbreviations

SCI: Spinal cord injury
qRT-PCR: Quantitative reverse transcription polymerase chain reaction
DAMPs: Damage-associated molecular patterns
HMGB1: High mobility group box 1
RAGE: Receptor for advanced glycation end products
NF-κB: Nuclear factor-kappa B
LFB: Luxol fast blue
BBB: Basso-Beattie-Bresnahan
RHI: Rump-height index
