# Supplementary figures and images for: Inhibiting HMGB1-RAGE axis prevents pro-inflammatory macrophages/microglia polarization and affords neuroprotection after spinal cord injury
Source: J Neuroinflammation. 2020 Oct 9;17:295. doi: 10.1186/s12974-020-01973-4 (PMC7547440; doi:10.1186/s12974-020-01973-4)

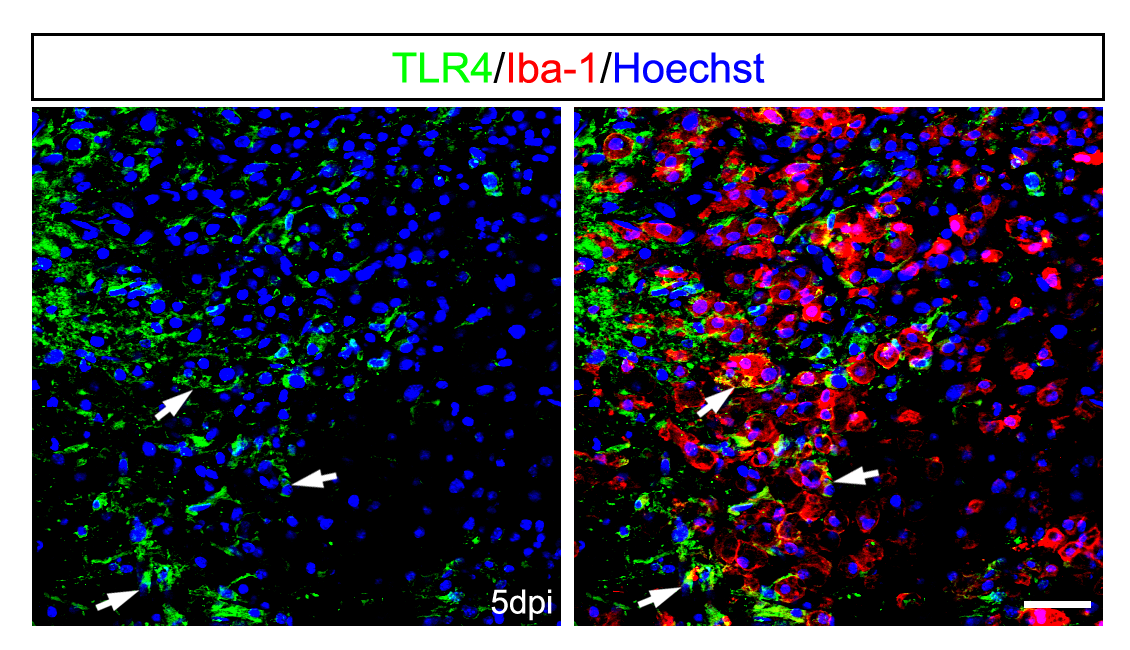

Supplement: Supplementary file 1 — Additional file 1: Figure S1. Double-staining of Iba-1/TLR4 at 5dpi. Note that about 17% TLR4-positive cells were Iba-1 positive. Scale bar = 50 μm. [file 12974_2020_1973_MOESM1_ESM.tif]

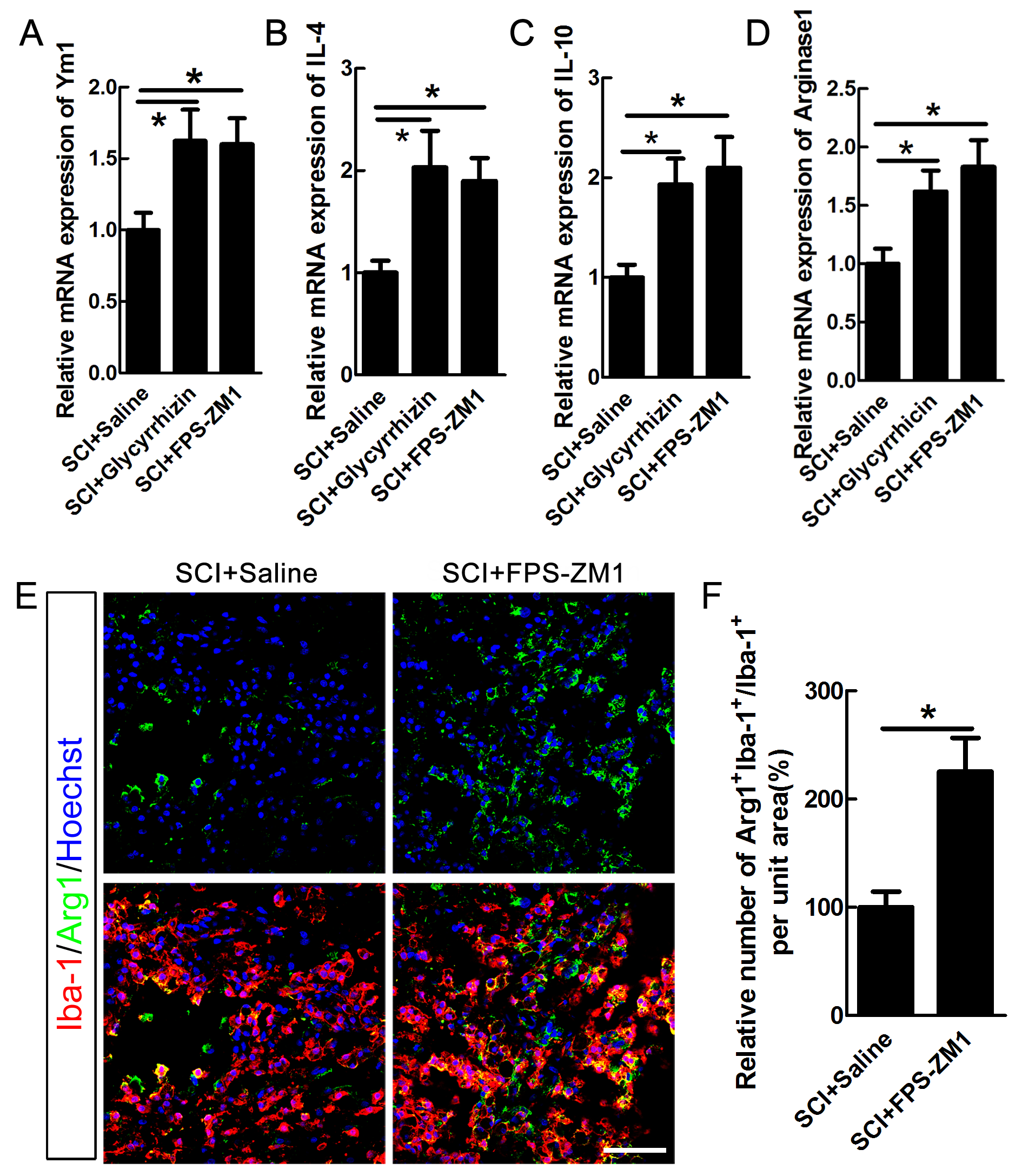

Supplement: Supplementary file 2 — Additional file 2: Figure S2. Inhibiting HMGB1 or RAGE increased the numbers of anti-inflammatory macrophages/microglia after SCI. Quantification of anti-inflammatory-associated mRNA transcripts of Arginase1 (A), Ym1 (B), IL-4 (C), IL-10 (D) in saline-, FPS-ZM1- or Glycyrrhizin-treated rats at 14 dpi. Results are presented as mean ± SEM of n=3, *P<0.05. (E) Double-staining of Arginase1 and Iba-1 in saline- or FPS-ZM1-treated rats at 14 dpi. Scale bar = 80 μm. (F) Quantification of the numbers of Arginase1-positive microglia/macrophages in lesion epicenter. Results are presented as mean ± SEM. N = 5/group, *P<0.05. [file 12974_2020_1973_MOESM2_ESM.tif]

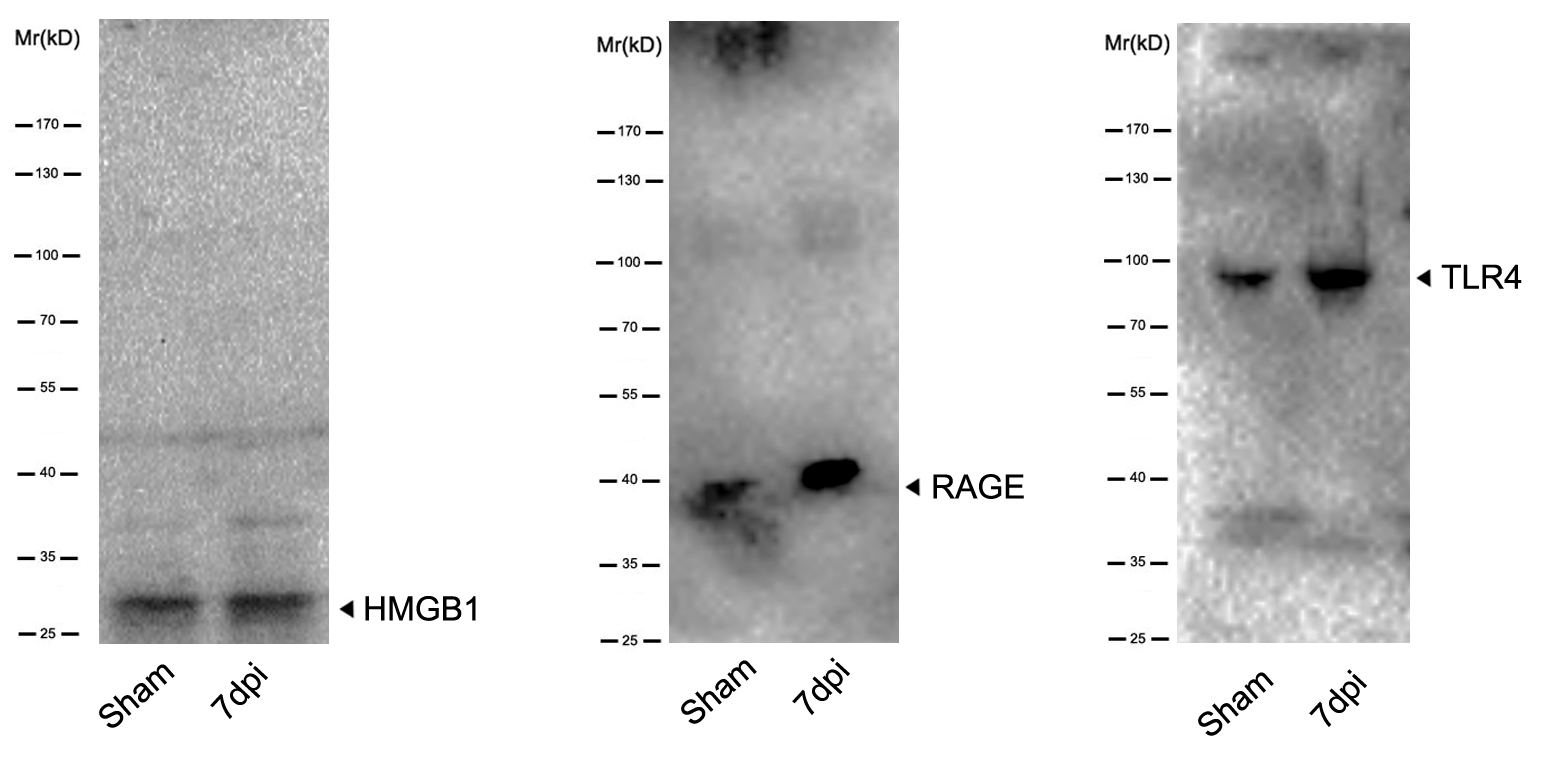

Supplement: Supplementary file 3 — Additional file 3: Figure S3. Western blot of HMGB1, RAGE, and TLR4. The specificity of antibodies of HMGB1, RAGE and TLR4 was verified by western blot. Note that the antibodies (HMGB1, RAGE, and TLR4) recognize only one antigen respectively, and each band is of the appropriate molecular weight. [file 12974_2020_1973_MOESM3_ESM.tif]

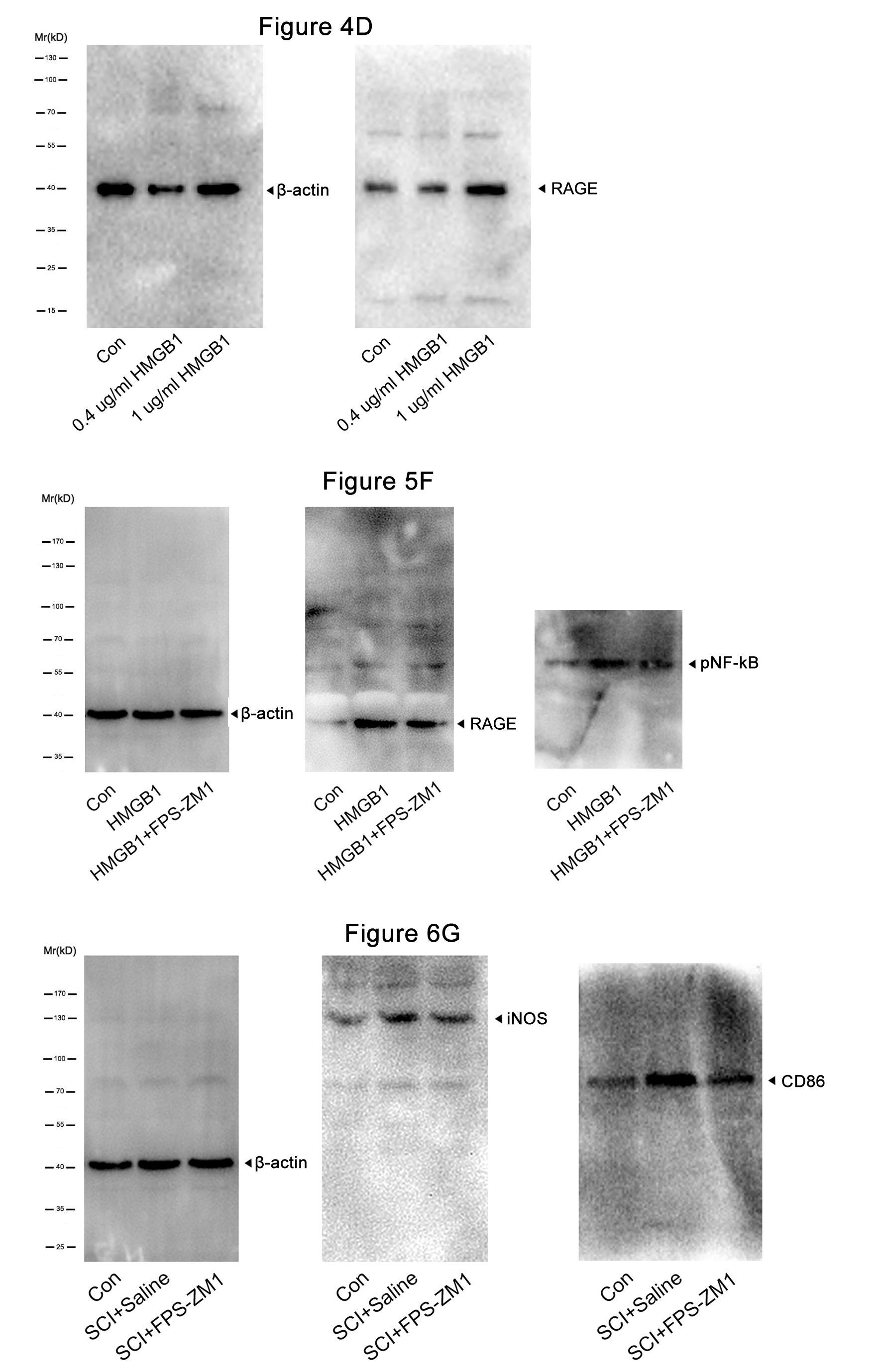

Supplement: Supplementary file 4 — Additional file 4: Figure S4. Full gels of RAGE, pNF-κB, iNOS, CD86 and β-actin. [file 12974_2020_1973_MOESM4_ESM.tif]
